# Visualizing Epigenetics: A Review of Microscopy Techniques for Investigating DNA Methylation Patterns, Chromatin Structure, and Gene Expression

**DOI:** 10.1093/mam/ozaf017

**Published:** 2025-03-28

**Authors:** Anna-Lee C Thompson, Judith L M Wopereis, Yonas I Tekle, Laura A Katz

**Affiliations:** Department of Biological Sciences, Smith College, 44 College Ln, Northampton, MA 01063, USA; Department of Biological Sciences, Smith College, 44 College Ln, Northampton, MA 01063, USA; Department of Biology, Spelman College, 350 Spelman Ln SW, Atlanta, GA 30314, USA; Department of Biological Sciences, Smith College, 44 College Ln, Northampton, MA 01063, USA; Program in Organismic and Evolutionary Biology, University of Massachusetts Amherst, 300 Massachusetts Ave, Amherst, MA 01003, USA

**Keywords:** epigenetics, fluorescence imaging, histone modifications, imaging

## Abstract

Microscopy approaches are frequently used to decipher the localization and quantify the abundance of biologically relevant molecular targets within single cells. Recent research has applied many optical imaging techniques to specifically visualize epigenetic modifications, the mechanisms by which organisms control gene expression in response to environmental factors. While many molecular and omics-based approaches are used to understand epigenetic mechanisms, imaging approaches provide spatial information that supplies greater context for discerning function. Thus, labeling approaches have been developed to quantify and visualize epigenetic targets using various fluorescence microscopy, electron microscopy, and super-resolution microscopy techniques. Here, we synthesize information about microscopy methods that enable visualization of epigenetic marks including DNA methylation, histone modifications, and localization of RNAs, which provide insights into mechanisms involved in chromatin remodeling and gene expression. The ability to determine how and where specific epigenetic marks manifest structurally and functionally in cells demonstrates the power of microscopy in aiding our understanding of epigenetic processes.

## Introduction

**Fig. 1.**
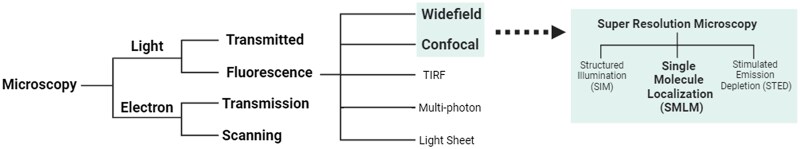
Graphical representation of microscopy techniques with applications to visualize epigenetic targets. Bolded and enlarged techniques are discussed in this review. Super-resolution methods can be adapted from widefield, confocal, or total internal reflection (TIRF) microscope systems (Lelek et al., 2021). Types of FM techniques listed are from Wang and Lai (2021) and Thorn (2016), and types of SRM from Lelek et al. (2021).

First developed in the 16th century, microscopy has since been used to examine the morphological features and miniscule structures of biological samples (Wills, 2018). Transmitted light microscopy was the first and most simple technique created, whereby a sample illuminated by a transmitted beam of light is magnified through a glass lens (Holgate & Webb, 2003). Following the advancement of transmitted light microscopy, numerous microscopy developments have improved resolution, contrast, and image quality. The most notable of these developments being fluorescence-based microscopy techniques as well as electron microscopy (EM), which uses a beam of electrons for illumination instead of light (Fig. 1). Transmitted light, fluorescence, electron, and other forms of microscopy within these umbrella terms have been leveraged to examine countless targets across biology, biochemistry, and other fields within the life sciences.

Only now, with the recent surge in epigenetics “hype” (Deichmann, 2016), have these microscopy techniques been applied in an attempt to visualize epigenetics (Weinhold, 2006). There are three different epigenetic targets or “marks” that regulate aspects of chromatin structure and transcription initiation (Casadesús & Noyer-Weidner, 2013). The first is DNA methylation, an epigenetic mark that causes negative regulation of gene expression by inhibiting transcription and promoting chromatin condensation (Moore et al., 2013). The second is histone modifications, which are chemical changes that can either promote or inhibit gene expression by reversibly altering the proteins around which DNA wraps to form chromatin (Mariño-Ramírez et al., 2005). The third is RNAs (mRNAs, lncRNAS, rRNA, etc.), gene products that determine protein production or are functional otherwise (non-protein-coding) and play a crucial regulatory role in gene expression and genome organization (Morris & Mattick, 2014).

These epigenetic marks and the mechanisms associated with them have been predominantly studied *via* next generation sequencing techniques. For example, (1) bisulfite sequencing (Bi-seq), methylated DNA immunoprecipitation sequencing (MeDIP-seq), and methylation-sensitive restriction enzyme sequencing (MRE-seq) are used to sequence DNA methylation, (2) chromatin immunoprecipitation sequencing (ChIP-seq) detects histone modifications, and (3) RNA sequencing methods can be modified to detect long non-coding RNAs (lncRNAs) and other RNAs (Table 1; Bell & Spector, 2011). While these techniques may be helpful in understanding the global abundance and variation of epigenetic marks, they provide little information about the spatial distribution of epigenetic targets along DNA or their localization in relation to chromatin and other structures. By employing microscopy, both the abundance and spatial location of epigenetic marks can be detected to further characterize their structure and function.

**Table 1.** Overview of Different Types of Sequencing and Microscopy/Labeling Techniques to Visualize DNA Methylation, Histone Modifications, and RNAs.

Epigenetic Mark	Sequencing Techniques (Bell & Spector, 2011)	Microscopy/Labeling Techniques
DNA methylation	Bi-seq, MeDIP-seq, MRE-seq	EM immunolabeling, methylation-specific FISH, EVA, spatial omics, methyl-CpG-binding domain (MBD)-based sensors
Histone modifications	ChIP-seq	FLIM, FRET, FCS, SRM, ISH-PLA, EVA, spatial omics, spatial-CUT&Tag, histone modification reader domain (HMRD)-based sensors
RNAs	RNA-seq, lncRNA-seq	FISH, RNA-FISH, smFISH, M-FISH, EM-ISH, SRM, EM immunolabeling, spatial omics, ExFISH

In this review, we examine how chemical probes, fluorescence microscopy (FM), EM, and other approaches are employed to visualize DNA methylation, histone modifications, and RNAs. Specifically, we exemplify how visualizing these epigenetic targets helps to elucidate their role in epigenetic mechanisms (e.g., chromatin remodeling and gene expression). Microscopy techniques are versatile, provide more information than current sequencing-based techniques alone, and enable epigenetic processes to be visualized by illuminating the epigenetic marks involved in genome organization and gene expression.

## Epigenetic Marks in Relation to Chromatin Structure

Epigenetic marks are largely responsible for chromatin remodeling mechanisms, which play critical roles in the regulation of all genes. More specifically, DNA methylation and histone acetylation status contribute to the condensation or expansion of chromatin (Albini et al., 2019). Visualizing DNA methylation and histone modifications in relation to chromatin structure is necessary for determining mechanisms of action and confirming or challenging already preconceived functions. For example, Masiello and Biggiogera (2017) characterize DNA methylation using EM by showing the distribution of methylation patterns in relation to chromatin structure. To visualize the ultrastructural localization of 5-methylcytosine (5mC) on DNA using TEM, they used immunolabeling with an anti-5mC antibody, a 6-nm gold-conjugated secondary antibody, along with stains for nucleic acids (Masiello & Biggiogera, 2017). Masiello and Biggiogera (2017) hypothesized that they would find more gold particles associated with regions of highly condensed chromatin near the nuclear envelope, as it is generally understood that DNA methylation promotes chromatin condensation. However, their findings show a greater abundance of gold antibodies toward the edge of the heterochromatin with concentration decreasing gradually approaching the nuclear envelope (Masiello & Biggiogera, 2017). This contradicts the implications of the known function of 5mC (i.e., that heterochromatin is more methylated) and suggests that more research is needed to confirm the role of 5mC and its associations with chromatin structure.

One possible explanation for this pattern is that as chromatin becomes more and more condensed, it is harder for the anti-5mC antibody to access the 5mC sites, which could result in reduced or no staining. If this is the case, then new EM labeling techniques aside from immunolabeling need to be developed in order to gain a more accurate approach. Despite this, this initial protocol highlights the potential of using EM to investigate the relationship between chromatin structure, DNA methylation, and other molecular interactions involved in chromatin organization.

**Fig. 2.**
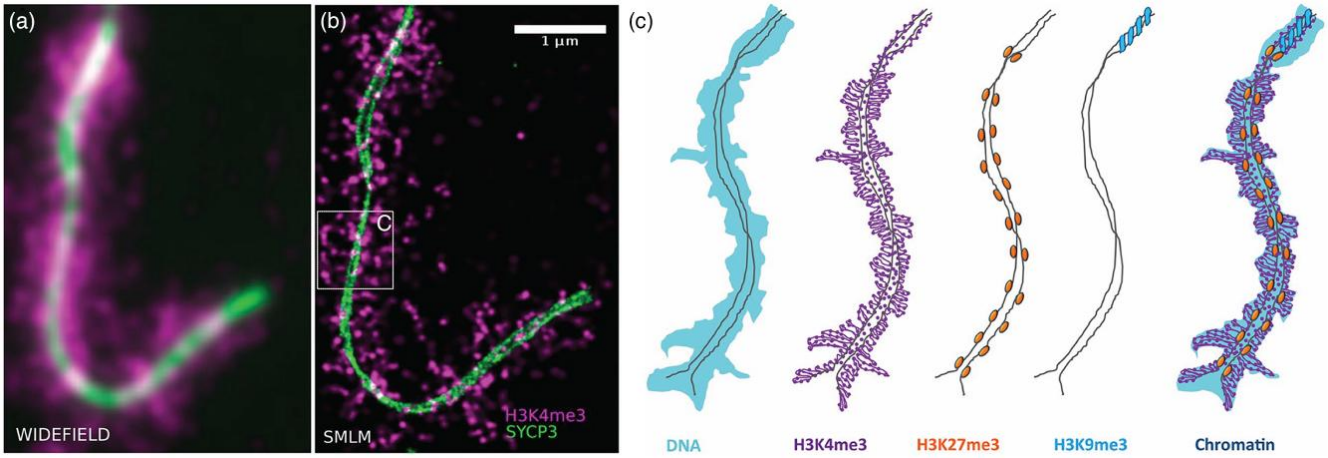
Visualization of histone modifications (H3K4me3) using widefield FM versus super-resolution SMLM and proposed histone modification patterns found along pachytene chromosomes forming the SC. A widefield fluorescence image of the SC with the lateral element of the SC, SYCP3, and the histone modification H3K4me3 staining (**a**). A SMLM image depicting the same staining with increased resolution (**b**). The patterns of histone modifications H3K4me3, H3K27me3, and H3K9me3 overlaid with DNA found along the SC (**c**). Adapted from Fig. 5 and 6 in Prakash et al. (2015) with permission.

Smiliarly, visualizing the localization of histone modifications in relation to chromatin structure can reveal the function of specific modifications, which is particularly useful for studying epigenetic changes throughout an organism's life or during key cellular events such as meiotic recombination. Prakash et al. (2015) exemplify this by capturing the first-ever single-molecule resolution images of histone modifications and chromatin in pachytene chromosomes that form the synaptonemal complex (SC) to examine if certain histone modifications are associated with meiotic recombination. They use super-resolution microscopy (SRM), a technique in which the diffraction barrier is reduced to acheive enhanced resolution compared to widefield fluorescence (Fig. 2a and 2b) (Schermelleh et al., 2019). Prakash et al. (2015) apply a subtechnique called single-molecule localization microscopy (SMLM), which involves computational rendering of an image after single-molecule detection and localization (Lelek et al., 2021). In this study, they used DNA dyes and fluorescently tagged antibodies targeting three specific histone modifications: the trimethylation of histone H3 at Lys 4 (H3K4me3), the trimethylation of histone H3 at Lys 27 (H3K27me3), and the trimethylation of histone H3 at Lys 9 (H3K9me3; Prakash et al., 2015). H3K4me3 is associated with recombination sites, and H3K27me3 and H3K9me3 are associated with depleted recombination sites (Prakash et al., 2015). Using these staining and microscopy techniques, Prakash et al. (2015) were able to decipher the locations of these histone modifications along pachytene chromosomes and found that they had distinct structural patterns along the SC (Prakash et al., 2015). H3K4me3 was found to extend outward in loop structures from the SC, H3K27me3 was found in period clusters along the SC, and H3K9me3 was associated with the centromeric region at the end of the chromosome (Fig. 2c; Prakash et al., 2015).

Altogether, Prakash et al. (2015) were able to describe the structure of pachytene chromosomes as characterized by epigenetic marks on chromatin during meiotic recombination. The unique spatial information gained from the use of fluorescent SMLM helped further define the function of these histone modifications. For example, H3K27me3 was found periodically and symmetrically on either side of the SC, which suggests that it could be associated with specific parts of the genome, or is integral to supporting the structure of the SC itself (Prakash et al., 2015). This demonstrates how visualizing localization and distribution helps to discern the function of specific histone modifications or other epigenetic marks during cellular events.

Another relevant tool for analyzing chromatin structure is fluorescence lifetime imaging coupled with Förster resonance energy transfer (FLIM-FRET). FRET is a mechanism in which one fluorophore transfers energy to a different fluorophore without emitting a photon (Shrestha et al., 2015). Since this phenomenon is distance dependent, FRET efficiency can be used to probe the proximity of two fluorophores (Liu and Irudayaraj, 2019). This is extremely useful for studying the condensation state of chromatin, as FRET efficiency will be greater in highly condensed heterochromatin and lower in loose euchromatin. FLIM microscopy is one method that can be used to calculate FRET efficiency as a means of investigating chromatin architecture. For example, Levchenko et al. (2021), Lou et al. (2019), and Zhang et al. (2021) use FLIM-FRET to measure DNA compaction, gene activity, and chromatin changes as a result of stimuli (e.g., double-stranded breaks, drugs, etc.).

Another useful method for investigating molecular machinery involved in chromatin remodeling and gene expression is fluorescence correlation spectroscopy (FCS), a biophysical technique used to measure fluorescence intensity changes caused by molecular interactions (Macháň & Wohland, 2014). FCS is primarily employed to analyze diffusion, kinetics, and binding events, making it particularly valuable in the field of epigenetics (Elson, 2011). Specifically, FCS can be used for studying protein–protein interactions, chromatin organization, chromatin binding, and protein–DNA interactions (Macháň & Wohland, 2014). Cui et al. (2013) highlight the capacity of FCS to study epigenetics through their use of the technique to probe interactions between methylated DNA and methyl-CpG-binding domain protein 3 (MBD3), a protein that maintains gene repression by binding to methylated DNA. The first study to examine these dynamics at single-molecule sensitivity in live cells, Cui et al. (2013) demonstrate that during hypoxia (stress), the MBD3 proteins detach from methylated DNA, allowing DNA demethylation to occur, eventually leading to cell death. The ability to closely analyze specific protein and DNA interactions using FCS or other techniques is relevant for applications within epigenetics.

## Gene Expression

Epigenetic processes regulate gene expression, and therefore, imaging RNAs provides a direct method to measure the genes being expressed in response to epigenetic changes or other cellular events. The visualization of specific RNAs can highlight what genes are being expressed, elucidate the role of non-protein coding RNAs, and illustrate how exposure to environmental factors might alter expression. RNAs are commonly labeled using *in situ* hybridization (ISH), in which complementary aptamers are annealed to target sequences (Table 2). For example, fluorescence *in situ* hybridization (FISH), a technique that visualizes a target nucleic acid sequence by hybridizing a fluorescently labeled complementary strand, is widely applied (Shakoori, 2017). RNAs, specifically, can be labeled *via* RNA-FISH, where the fluorescently labeled complementary DNA or RNA oligonucleotide strand is hybridized to a target RNA sequence (Chen et al., 2015; Young et al., 2020). RNA-FISH has also been used to image functional lncRNAs and other RNAs, which are known to play an important role in epigenetic processes such as chromatin remodeling and transcription regulation (Kaikkonen et al., 2011). When RNA-FISH is coupled with other immunofluorescent markers and DNA stains, the co-localization and abundance of epigenetic markers can reveal unknown functions (Baker, 2012; Hinten et al., 2016).

**Table 2.** Summary of ISH Techniques to Visualize Epigenetic Targets.

Technique	Molecular Target(s)	Types of Microscopy	Source
FISH	DNA	FM, SRM	Shakoori 2017
RNA-FISH	RNA; mRNA, lncRNA	FM, SRM, ExM	Young et al., 2020
Single-molecule FISH (smFISH)	RNA; mRNA, lncRNA, viral RNA genomes, rRNA	FM, SRM, ExM	Haimovich and Gerst (2018)
Multiplex FISH (M-FISH)	DNAs, RNAs	FM, SRM, ExM	Shakoori (2017)
Electron microscopy-ISH (EM-ISH)	DNA, RNA	EM	Cmarko and Koberna (2007)
Methylation-specific FISH (MeFISH)	DNA methylation	FM, SRM	Li et al. (2013)
ISH-proximity ligation assay (ISH-PLA)	Histone modifications	FM, SRM	Gomez et al. (2013)
Epigenetic visualization assay (EVA)	DNA methylation, histone modifications	FM, SRM	Kint et al. (2021)
Expansion FISH (Ex-FISH)	RNA; mRNA, lncRNA	FM, SRM, ExM	Chen et al. (2016)

**Fig. 3.**
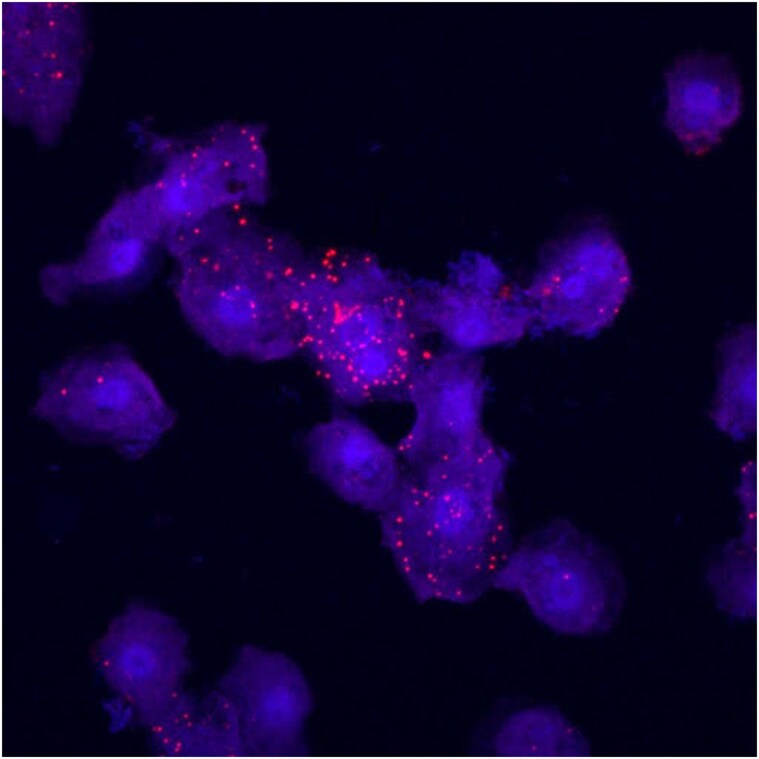
Unpublished confocal images of *Cochliopodium* cells labeled with a Pch2 RNA-FISH probe (red) and a DNA stain (blue). Pch2 has greater expression in cells undergoing fusion. Nuclei in unfused cells are ~6 µm. Images from study by Tekle et al. (2020) with permission.

RNA-FISH is especially useful in quantifying the presence of gene products among samples that lack robust reference genomes or genes that have low enough expression that they are hard to detect using transcriptomic methods (Tekle et al., 2020). Tekle et al. (2020) use this advantage of RNA-FISH to visualize meiosis-specific gene transcripts in order to characterize the sexual development of *Cochliopodium minus*, an amoeba in the Amoebozoa clade. Genetic studies have observed that all studied lineages of Amoebozoa possess sex-related genes including meiosis-specific genes (Tekle et al, 2017, 2020). However, sex has only been observed in a small number of Amoebozoa species, indicating that some species may not have observable sexual stages (Tekle et al., 2020). To examine the sexual development of *Cochliopodium*, Tekle et al. (2020) use RNA-FISH for some of the meiosis-specific gene mRNA transcripts including Pch2, Dmc1, Mer3, and Kem1. They saw that many of the meiosis-specific genes, and most prominently, Pch2, were among upregulated genes in fused cells (Fig. 3). Based on this association, they were able to conclude that cellular fusion is a sexual stage in *Cochliopodium* (Tekle et al., 2020). This is particularly interesting because Pch2 was not among the differentially expressed genes in their analyses even though the Pch2 RNAs localized specifically in fused cells (with increased prevalence and intensity of signal), demonstrating that meiosis was likely occurring at fusion (Tekle et al., 2020). RNA-FISH can also be used to measure the expression of RNAs associated with epigenetic modifications (e.g., during development or following environmental stressors). For example, Yue et al. (2014) use RNA-FISH to visualize the Xist lncRNA involved in epigenetic regulation of X-chromosome inactivation in mammals.

Along with RNA-FISH, other types of FISH can be applied to visualize candidate epigenetic genes (mRNA transcripts and epigenetic regulatory RNAs), one of which being single-molecule FISH (smFISH). smFISH uses up to 48 fluorescent oligonucleotides, which all bind to the same RNA target molecule (Shakoori, 2017). Using multiple fluorescent probes for the same target allows for reduced noise and amplified signal of RNAs that may be expressed at low rates (Pichon et al., 2018). smFISH samples can also be imaged using super-resolution microscopes to achieve greater resolution, which is necessary for examining highly concentrated RNAs (Pichon et al., 2018). Moreover, smFISH has been adapted to simultaneously visualize multiple RNA targets in the same sample using different fluorophores, a technique known as multiplex FISH (M-FISH) (Shakoori, 2017). M-FISH is a valuable ttechnique for investigating epigenetic changes because, often, there are a number of RNAs and epigenetic marks involved in expression changes.

Another challenge is that many RNAs occur at high concentrations and exist in complex environments, which requires enhanced resolution to discern precise spatial organization. In this case, increased resolution can be achieved by using expansion microscopy (ExM), a technique in which samples are physically expanded *via* hydrogel swelling (Cho et al., 2018). For even greater resolution, there is also some evidence that RNA can be visualized using EM after staining with terbium citrate as proposed by Biggiogera and Masiello (2017), which would also provide relevant data regarding gene expression. Electron microscopy *in situ* hybridization (EM-ISH) is also an option to track DNA or RNA sequences at high resolution using TEM or SEM (Cmarko & Koberna, 2007).

Another important factor for analyzing gene expression is investigating RNA–protein interactions, which can be explored using stimulated emission depletion (STED) microscopy to map nuclear proteins (Sarmento et al., 2018). This is done by performing super-resolution STED microscopy on nuclei that have both RNA labeling and protein immunofluorescence staining to examine colocalizations (Dumbović et al., 2021). For example, Dumbović et al. (2021) use this approach to analyze lncRNA–protein aggregation and its possible function and downstream affects on cancer. Probing RNA–protein interactions using STED enables greater understanding of subnuclear structure–function relationships involved in gene expression and epigenetic processes.

## Gene and Sequence Specificity

Microscopy techniques allow for the examination of epigenetic marks at specific genomic locations with gene- or sequence-level resolution. This includes exclusively imaging DNA methylation patterns of specific genes and satellite repeat regions, or as mentioned previously, the labeling of only target RNAs *via* FISH. This is relevant because the abundance of epigenetic marks, such as DNA methylation at specific loci, can be associated with certain diseases or predispositions, whereas many sequencing-based techniques only provide global- or population-level information.

Several chemical probe labeling techniques provide greater specificity, enabling the characterization of epigenetic marks along a particular chain of nucleotides. One such approach is methylation-specific FISH (MeFISH), a method proposed by Li et al. (2013) to visualize DNA methylation patterns on specific satellite repeat sequences. The methylation of satellite repeat regions contributes to the formation of heterochromatin and chromosome structures (Shatskikh et al., 2020). Furthermore, hyper- or hypomethylation of certain satellite repeat regions can occur as a result of environmental factors and is associated with specific cancerous, neurodegenerative, psychiatric, and autoimmune diseases (Pogribny & Beland, 2009).

**Fig. 4.**
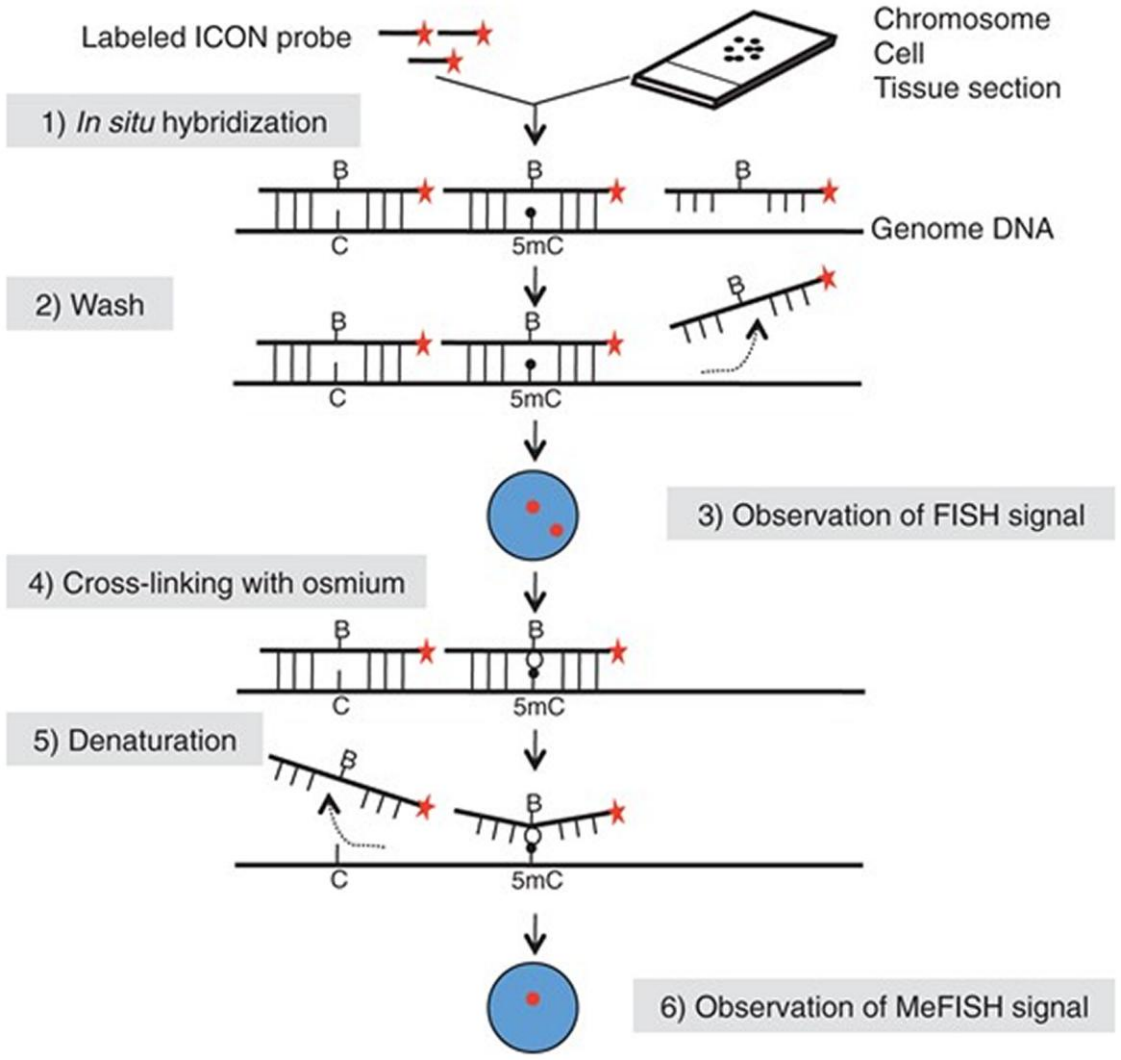
MeFISH procedure depicting labeling of DNA methylation and hydroxymethylation using ICON probes. The probes are first hybridized to target satellite repeat sequences and following washes, only probes bound to cytosine are left. Then, the sample undergoes crosslinking with osmium, binding the ICON probes to methylated cytosines (5mC) and hydroxymethylated cytosine (5hmC). The probes are then denatured and only probes bound to 5mC and 5hmC remain. Figure from Li et al. (2013).

In the development of MeFISH, Li et al. (2013) create “ICON” probes by combining the typical FISH method (i.e., fluorescently labeled complementary sequences) with bipyridine-containing nucleic acids that only bind to 5mC when treated with osmium. Fluorescently labeled ICON probes are hybridized to satellite repeat regions of DNA, and then, the sample undergoes crosslinking with osmium and denaturing, leaving behind only the methylated (5mC) and hydroxymethylated (5hmC) forms of cytosine (Fig. 4; Li et al., 2013). Specificity toward both 5mC and 5hmC is useful in the case when you expect a particular methylation/hydroxymethylation status and can confirm with antibodies for the two targets (Li et al., 2013). Sequence-specific labeling approaches are powerful because they allow for the methylation of specific DNA sequences to be detected in comparison with all of the DNA in a cell or chromosome.

More recently, there have also been advances in fluorescence imaging of gene-specific epigenetic markers using *in situ* hybridization–proximity ligation assays (ISH-PLAs) and epigenetic visualization assays (EVAs) (Cui & Irudayaraj, 2017; Kint et al., 2021). Gomez et al. (2013) describe the first-ever visualization of histone modifications at specific gene loci within single cells using ISH-PLA. ISH-PLA consists of a combination of *in situ* hybridization and proximity ligation assays, which are frequently used to detect and quantify proteins, protein–protein interactions, and protein modifications (Faron-Górecka et al., 2019). Visualizing histone modifications at specific gene loci is useful in order to determine their role in epigenetic events.

**Fig. 5.**
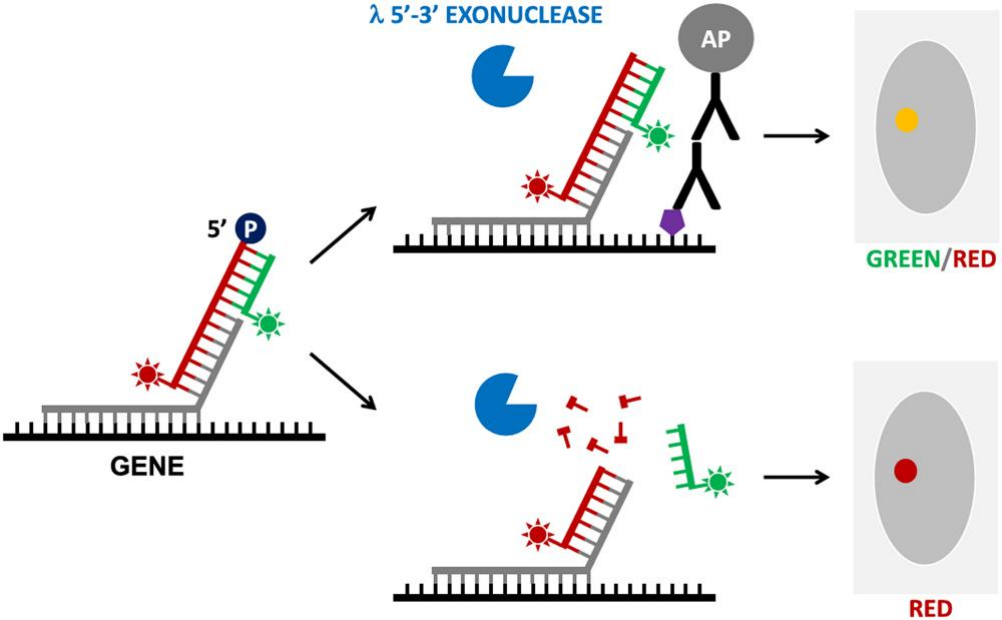
Scheme depicting the EVA probe labeling procedure. An overhanging 5′ phosphorylated (P) fluorescently labeled oligonucleotide (red strand) and a shorter complementary probe (green strand) bind to the target gene. The alkaline phosphatase (AP) antibody binds to the target epigenetic mark (purple pentagon) and removes the phosphate group from the probe thereby protecting it from λ-exonuclease (blue sector) degradation (top) showing both green and red fluorescence during imaging (yellow circle). Probes that do not have the epigenetic mark and remain phosphorylated will be degraded by λ-exonuclease and will lose a fluorescent tag leaving only red fluorescence during imaging (red circle). Figure from graphical abstract of Kint et al. (2021).

The EVA, developed by Kint et al. (2021), also achieves visualizations of the epigenetic marks of individual gene alleles within a single cell. This method utilizes both a 5′ phosphorylated oligonucleotide labeled with two fluorescent tags that binds to a target gene sequence and an alkaline phosphatase-linked antibody that binds to the specific epigenetic mark (Fig. 5; Kint et al., 2021). If the epigenetic mark is present at the target gene, then the alkaline phosphatase will remove the phosphate group from the probe leaving the two fluorescent tags intact (Kint et al., 2021). Then, the sample is treated with λ-exonuclease, an enzyme that degrades phosphorylated double-stranded DNA, and the probes with the epigenetic mark will remain untouched, while the probes bound to the gene lacking the epigenetic mark will have one fluorescent tag removed (Kint et al., 2021). The signal of each fluorophore can then be quantified to determine the density of epigenetic marks (Kint et al., 2021). This approach is particularly beneficial because it can be done alongside RNA-FISH displaying the relationship between epigenetic states and gene transcription (Kint et al., 2021). The EVA, ISH-PLA, and MeFISH methods demonstrate the versatility of microscopy techniques through their capability of examining both global populations like many modern sequencing approaches as well as enabling exclusive detection of epigenetic marks at specific sequences or gene loci.

Numerous spatial omics methods that map high-throughput sequences to their original location within a tissue sample have also been applied to visualize epigenetic targets. For example, Deng et al. (2022b) use spatial-ATAC-seq to resolve chromatin accessibility in mouse embryos and identify cell-type-specific chromatin regulatory elements. Other techniques such as cleavage under targets and tagmentation (CUT&Tag) and spatial-CUT&Tag enable further specificity by targeting specific sequences or epigenetic marks (Kaya-Okur et al., 2019). Deng et al. (2022a) and Kaya-Okur et al. (2019) use CUT&Tag to isolate regions of the genome that are associated with histone modifications, RNA polymerase II, and transcription factors. Omics techniques such as CUT&Tag, spatial-CUT&Tag, spatial-ATAC-seq, and epigenomic MERFISH enable spatial profiling of gene expression, chromatin accessibility, and histone modifications essential for understanding epigenetic processes (Lu et al. 2022).

Recent studies have explored methods to combine omics data and fluorescence imaging within the same sample. However, this is challenging to accomplish because it requires integration across multiple modalities and data types (Yang et al., 2021). Yang et al. (2021) illustrate the value of merging sequence and imaging data for studying epigenetics by developing a computational approach that integrates single-cell RNA sequence data and chromatin fluorescence images. Emerging techniques such as Deterministic Barcoding in Tissue sequencing plus (DBiTplus) and HyperSeq offer additional strategies for integrating imaging and sequence data. DBiTplus combines sequence-based spatial transcriptomics with image-based multiplexed protein immunofluorescence profiling, enabling simultaneous acquisition of both gene expression and protein data from a single sample (Enninful et al., 2024). Similarly, HyperSeq also uses spatial transcriptomics, but instead merges it with hyperspectral imaging, a microscopy technique that detects biomolecular autofluorescence (Xie et al., 2024). By integrating sequence data with spectral, morphological, and/or localization information, DBiTplus and HyperSeq provide insights useful for cell-type identification, gene expression analysis, mechanistic pathway investigation, among other applications (Enninful et al., 2024; Xie et al., 2024). These advances highlight the benefits of integrating image and sequence data, particularly in enhancing spatial context for studying biological processes including epigenetics.

## Limitations and Opportunities

While visualization-based techniques generally provide more information about epigenetic marks than other techniques, they also have some limitations and opportunities for improvement. Some of the most imminent being resolution limitations, fixed versus live samples, and target accessibility. However, there are many examples of techniques and applications that attempt to combat these limitations as described below.

### Target Accessibility and Availability

One limitation to labeling methods is that many epigenetic targets are difficult to label or occur at high concentrations. For example, many RNAs are highly expressed and thus, it can be difficult to draw conclusions when comparing relationships between RNA expression and another epigenetic mark. There are also many challenges associated with attempting to image DNA methylation because of high nuclear density *in situ* and reduced accessibility to antibodies due to the embedded position of cytosine within the double helix (Cui & Irudayaraj, 2017). Due to this, there are limited approaches to labeling DNA methylation in an accurate manner. One approach to tackling this challenge is using molecules already found in biological systems whose function include binding the desired epigenetic mark. For example, methyl-CpG-binding domain (MBD) proteins are inherently attracted to DNA methylation sites and contribute to chromatin formation (Cui & Irudayaraj, 2017). Leveraging the chemical structure of MBDs and adapting them to incorporate fluorophores or other labels could solve the accessibility issues of methylated cytosines. The development of sensors based on naturally occurring proteins and regulatory elements can resolve some of the target accessibility problems associated with probe labeling and enable more options for live cell imaging.

### Fixed versus Live Samples

Many labeling techniques can only be performed on fixed samples, limiting applications for studying life cycles and responses to environmental stimuli. Fixed samples are generally preferred for many reasons, with one primary advantage being that the sample will not be affected by phototoxicity—damage to cells that occurs after intense exposure to light (Icha et al., 2017). Additionally, fixed samples last longer, provide a snapshot of a single event, and are required for many techniques in order to maintain the structure of the sample. For example, immunofluorescence, one of the most common labeling approaches, can only be performed on fixed samples, so other methods are needed to track epigenetic marks in live cells (Im et al., 2019). One way this can be combated is by using genetically encoded sensors in model organisms. An example of this is using reader domain-based sensors to image histone modifications in live cells. Histone modification reader domains (HMRDs) are naturally occurring regions on histones that attract “reader” proteins to bind to specific histone modifications (Musselman et al., 2012). Recombinant HMRDs can be used to create genetically encoded fluorescent sensors (Stepanov et al., 2022). This enables the visualization of histone modifications in live cells which could be used to measure the presence and abundance of certain modifications over time, during cellular events, or in response to a stressor/stimuli. Besides reader domain-based techniques, other genetically encoded antibody-based or FRET sensors, as well as SRM techniques, can be applied to visualize live cells (Stepanov, 2022).

### Resolution Limits

**Fig. 6.**
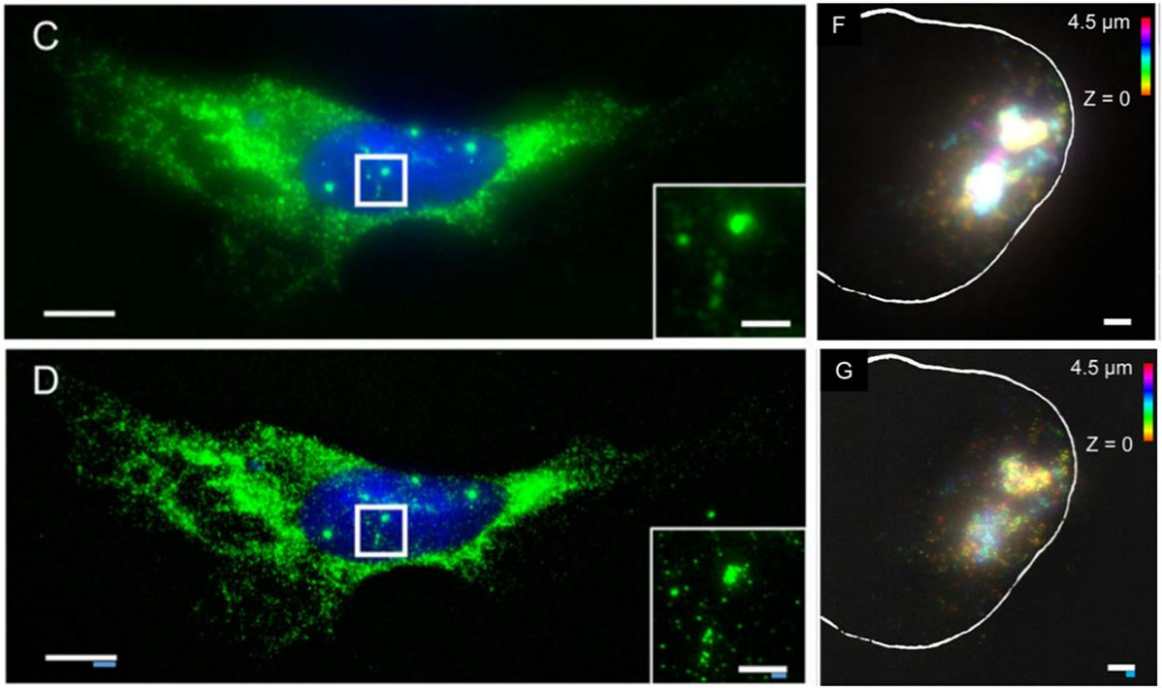
Images of smFISH labeled RNAs before and after ExFISH. Image of ACTB RNA (green) and DNA (blue) in a Hela cell before (**c**) and after ExM (ExFISH) (**d**). Image of Xist lncRNAs in an HEK293 cell before (**f**) and after expansion. (**c**, **d**) Scale bar = 10 *µ*m, zoom in scale bar = 2 *µ*m. (**f**, **g**) Scale bar = 2 *µ*m; different colors represent each *z* slice; white outline is the nuclear envelope. White scale bars are in pre-expansion units; blue scale bars are pre-expansion units divided by the expansion factor. Modified from Fig. 1 of Chen et al. (2016) with permission.

Lastly, resolution limits can pose drawbacks in acquiring accurate and comprehensive data. While EM techniques are able to achieve up to 0.23 nm resolution, light and FM techniques usually have a much higher resolution limit between 250 and 420 nm due to the diffraction of light (Huang et al., 2009; Penczek, 2010). However, SRM methods have been able to overcome the diffraction barrier to achieve resolutions as low as 20–50 nm (Huang et al., 2009). Another approach to improving resolution is expansion microscopy (ExM) (Chen et al., 2016). Chen et al. (2016) developed an approach called expansion FISH (ExFISH), combining ExM and FISH to image RNAs at increased resolution. They synthesized a small molecule that links RNAs to an ExM gel, which then undergoes swelling/expansion followed by FISH to label RNAs (Chen et al., 2016). In this study, they performed smFISH on postexpanded HeLa cells to visualize mRNAs transcribed from ACTB, a gene within the actin gene family, as well as the Xist lncRNAs involved in X-chromosome inactivation (Fig. 6) (Chen et al., 2016). Postexpanded samples were expanded by a factor of 3.3X which resulted in greater resolution and may be particularly useful for imaging on microscopes with higher diffraction limits (Chen et al., 2016). Multiplex FISH can also be performed after expansion to visualize multiple RNA targets (Chen et al., 2016).

Besides ExFISH, other innovative methods, such as SRM, multi-photon microscopy, minimal photon fluxes (MINFLUX), as well as combinatorial approaches like expansion-assisted iterative FISH (EASI-FISH), which combines multiplex FISH with ExM, can be applied to reduce resolution limits and more accurately visualize RNAs, gene expression, and epigenetic changes (Schmidt et al., 2021; Wang et al., 2021; Sinefeld et al., 2022). Additionally, overall improvements on microscopes themselves will minimize resolution limits and further science.

## Conclusion

While microscopy techniques have some limitations, they are still robust tools to accurately characterize epigenetic marks such as DNA methylation, histone modifications, and RNAs. Visualization of the spatial and temporal distribution of epigenetic marks aids in revealing their functions related to genome organization, gene expression, and other biochemical pathways. Additionally, microscopy techniques can achieve a level of specificity that sequence-based techniques frequently lack.

The power behind microscopy techniques lies in the ability to observe, understand, and analyze intricate structures and details that elucidate functions and relationships. Imaging allows for the entire picture to be seen, enabling researchers to decipher the relative abundance, spatial distribution across a sample, and variation under differing conditions. Spatial localization uncovers relationships that epigenetic marks have in relation to chromatin structure, gene expression, and specific genomic regions. Additionally, several methods allow multiple epigenetic, genetic, or structural targets to be examined simultaneously, further informing how epigenetic marks and other relevant molecules are interconnected. Altogether, microscopy techniques and advanced labeling approaches are dynamic strategies that illustrate how structure informs function and showcase the power of visualizing epigenetics.

## Availability of Data and Materials

The authors have declared that no datasets apply for this piece.

## Financial Support

This work was supported by grants from the National Science Foundation: DEB-2230391 and OCE-1924570 to L.A.K., and National Institutes of Health: 1R15GM116103-02 to Y.T.

## References

[ozaf017-B1] Albini S, Zakharova V & Ait-Si-Ali S (2019). Chapter 3—Histone modifications. In Epigenetics and Regeneration, Palacios D (Ed.), vol. **11**, pp. 47–72. Academic Press. 10.1016/B978-0-12-814879-2.00003-0

[ozaf017-B2] Baker M (2012). RNA imaging in situ. Nat Methods 9(8), 787–790. 10.1038/nmeth.2108

[ozaf017-B3] Bell JT & Spector TD (2011). A twin approach to unraveling epigenetics. Trends Genet 27(3), 116–125. 10.1016/j.tig.2010.12.005 21257220 PMC3063335

[ozaf017-B4] Biggiogera M & Masiello I (2017). Visualizing RNA at electron microscopy by terbium citrate. In Histochemistry of Single Molecules: Methods and Protocols, Pellicciari C & Biggiogera M (Eds.), pp. 277–283. Springer. 10.1007/978-1-4939-6788-9_21 28155162

[ozaf017-B5] Casadesús J & Noyer-Weidner M (2013). Epigenetics. In Brenner's Encyclopedia of Genetics (Second Edition), Maloy S & Hughes K (Eds.), pp. 500–503. Academic Press. 10.1016/B978-0-12-374984-0.00480-0

[ozaf017-B6] Chen KH, Boettiger AN, Moffitt JR, Wang S & Zhuang X (2015). Spatially resolved, highly multiplexed RNA profiling in single cells. Science 348(6233), aaa6090. 10.1126/science.aaa6090 25858977 PMC4662681

[ozaf017-B7] Chen F, Wassie AT, Cote AJ, Sinha A, Alon S, Asano S, Daugharthy ER, Chang J-B, Marblestone A, Church GM, Raj A & Boyden ES (2016). Nanoscale imaging of RNA with expansion microscopy. Nat Methods 13(8), 679–684. 10.1038/nmeth.3899 27376770 PMC4965288

[ozaf017-B8] Cho I, Seo JY & Chang J (2018). Expansion microscopy. J Microsc 271(2), 123–128. 10.1111/jmi.12712 29782656

[ozaf017-B9] Cmarko D & Koberna K (2007). Electron microscopy in situ hybridization: Tracking of DNA and RNA sequences at high resolution. Meth Mol Biol (Clifton, N.J.) 369, 213–228. 10.1007/978-1-59745-294-6_11 17656753

[ozaf017-B10] Cui Y, Cho I-H, Chowdhury B & Irudayaraj J (2013). Real-time dynamics of methyl-CpG-binding domain protein 3 and its role in DNA demethylation by fluorescence correlation spectroscopy. Epigenetics 8(10), 1089–1100. 10.4161/epi.25958 23974971 PMC3891690

[ozaf017-B11] Cui Y & Irudayaraj J (2017). Optical microscopy and spectroscopy for epigenetic modifications in single living cells. In Epigenetics and Gene Expression in Cancer, Inflammatory and Immune Diseases, Stefanska B & MacEwan DJ (Eds.), pp. 135–154. Springer New York. 10.1007/978-1-4939-6743-8_9

[ozaf017-B12] Deichmann U (2016). Epigenetics: The origins and evolution of a fashionable topic. Dev Biol 416(1), 249–254. 10.1016/j.ydbio.2016.06.005 27291929

[ozaf017-B13] Deng Y, Bartosovic M, Kukanja P, Zhang D, Liu Y, Su G, Enninful A, Bai Z, Castelo-Branco G & Fan R (2022a). Spatial-CUT&tag: Spatially resolved chromatin modification profiling at the cellular level. Science 375(6581), 681–686. 10.1126/science.abg7216 35143307 PMC7612972

[ozaf017-B14] Deng Y, Bartosovic M, Ma S, Zhang D, Kukanja P, Xiao Y, Su G, Liu Y, Qin X, Rosoklija GB, Dwork AJ, Mann JJ, Xu ML, Halene S, Craft JE, Leong KW, Boldrini M, Castelo-Branco G & Fan R (2022b). Spatial profiling of chromatin accessibility in mouse and human tissues. Nature 609(7926), 375–383. 10.1038/s41586-022-05094-1 35978191 PMC9452302

[ozaf017-B15] Dumbović G, Sanjuan X, Perucho M & Forcales S-V (2021). Stimulated emission depletion (STED) super resolution imaging of RNA- and protein-containing domains in fixed cells. Methods 187, 68–76. 10.1016/j.ymeth.2020.04.009 32360441

[ozaf017-B16] Elson EL (2011). Fluorescence correlation spectroscopy: Past, present, future. Biophys J 101(12), 2855. 10.1016/j.bpj.2011.11.012 22208184 PMC3244056

[ozaf017-B17] Enninful A, Zhang Z, Klymyshyn D, Zong H, Bai Z, Farzad N, Su G, Baysoy A, Nam J, Yang M, Lu Y, Zhang N, Braubach O, Xu M, Ma Z, & Fan R. (2024). Integration of imaging-based and sequencing-based spatial omics mapping on the same tissue section via DBiTplus. Res Sq. 10.21203/rs.3.rs-5398491/v1 PMC13541625 41540123

[ozaf017-B18] Faron-Górecka A, Szlachta M, Kolasa M, Solich J, Górecki A, Kusmider M, Zurawek D & Dziedzicka-Wasylewska M (2019). Chapter 10—Understanding GPCR dimerization. In Methods in Cell Biology, Shukla AK (Ed.), Vol. **149**, pp. 155–178. Academic Press. 10.1016/bs.mcb.2018.08.005 30616817

[ozaf017-B19] Gomez D, Shankman LL, Nguyen AT & Owens GK (2013). Detection of histone modifications at specific gene loci in single cells in histological sections. Nat Methods 10(2), 171–177. 10.1038/nmeth.2332 23314172 PMC3560316

[ozaf017-B20] Haimovich G & Gerst JE (2018). Single-molecule fluorescence in situ hybridization (smFISH) for RNA detection in adherent animal cells. Bio Protoc 8(21), e3070. 10.21769/BioProtoc.3070 PMC8342053 34532531

[ozaf017-B21] Hinten M, Maclary E, Gayen S, Harris C & Kalantry S (2016). Visualizing long noncoding RNAs on chromatin. Meth Mol Biol (Clifton, N.J.) 1402, 147–164. 10.1007/978-1-4939-3378-5_12 PMC5094191 26721489

[ozaf017-B22] Holgate JH & Webb J (2003). Microscopy| light microscopy and histochemical methods. In Encyclopedia of Food Sciences and Nutrition, 2nd ed., Caballero B (Ed.), pp. 3917–3922. Academic Press. 10.1016/B0-12-227055-X/00778-1

[ozaf017-B23] Huang B, Bates M & Zhuang X (2009). Super resolution fluorescence microscopy. Annu Rev Biochem 78, 993–1016. 10.1146/annurev.biochem.77.061906.092014 19489737 PMC2835776

[ozaf017-B24] Icha J, Weber M, Waters JC & Norden C (2017). Phototoxicity in live fluorescence microscopy, and how to avoid it. BioEssays 39(8), 1700003. 10.1002/bies.201700003 28749075

[ozaf017-B25] Im K, Mareninov S, Diaz MFP & Yong WH (2019). An introduction to performing immunofluorescence staining. Meth Mol Biol (Clifton, N.J.) 1897, 299–311. 10.1007/978-1-4939-8935-5_26 PMC6918834 30539454

[ozaf017-B26] Kaikkonen MU, Lam MTY & Glass CK (2011). Non-coding RNAs as regulators of gene expression and epigenetics. Cardiovasc Res 90(3), 430–440. 10.1093/cvr/cvr097 21558279 PMC3096308

[ozaf017-B27] Kaya-Okur HS, Wu SJ, Codomo CA, Pledger ES, Bryson TD, Henikoff JG, Ahmad K & Henikoff S (2019). CUT&Tag for efficient epigenomic profiling of small samples and single cells. Nat Commun 10(1), 1930. 10.1038/s41467-019-09982-5 31036827 PMC6488672

[ozaf017-B28] Kint S, Van Criekinge W, Vandekerckhove L, De Vos WH, Bomsztyk K, Krause DS & Denisenko O (2021). Single cell epigenetic visualization assay. Nucleic Acids Res 49(8), e43. 10.1093/nar/gkab009 33511400 PMC8096246

[ozaf017-B29] Lelek M, Gyparaki MT, Beliu G, Schueder F, Griffié J, Manley S, Jungmann R, Sauer M, Lakadamyali M & Zimmer C (2021). Single-molecule localization microscopy. Nat Rev Methods Primers 1(1), 1–27. 10.1038/s43586-021-00038-x PMC9160414 35663461

[ozaf017-B30] Levchenko SM, Pliss A, Peng X, Prasad PN & Qu J (2021). Fluorescence lifetime imaging for studying DNA compaction and gene activities. Light Sci Appl 10(1), 224. 10.1038/s41377-021-00664-w 34728612 PMC8563720

[ozaf017-B31] Li Y, Miyanari Y, Shirane K, Nitta H, Kubota T, Ohashi H, Okamoto A & Sasaki H (2013). Sequence-specific microscopic visualization of DNA methylation status at satellite repeats in individual cell nuclei and chromosomes. Nucleic Acids Res 41(19), e186. 10.1093/nar/gkt766 23990328 PMC3799461

[ozaf017-B32] Liu W & Irudayaraj J (2019). Understanding the dynamics and structure of epigenetic states with single-molecule fluorescence microscopy. Curr Opin Biomed Eng 12, 18–24. 10.1016/j.cobme.2019.08.010

[ozaf017-B33] Lou J, Scipioni L, Wright BK, Bartolec TK, Zhang J, Masamsetti VP, Gaus K, Gratton E, Cesare AJ & Hinde E (2019). Phasor histone FLIM-FRET microscopy quantifies spatiotemporal rearrangement of chromatin architecture during the DNA damage response. Proc Natl Acad Sci U S A 116(15), 7323–7332. 10.1073/pnas.1814965116 30918123 PMC6462080

[ozaf017-B34] Lu T, Ang CE & Zhuang X (2022). Spatially resolved epigenomic profiling of single cells in complex tissues. Cell 185(23), 4448–4464.e17. 10.1016/j.cell.2022.09.035 36272405 PMC9691621

[ozaf017-B35] Macháň R & Wohland T (2014). Recent applications of fluorescence correlation spectroscopy in live systems. FEBS Lett 588(19), 3571–3584. 10.1016/j.febslet.2014.03.056 24726724

[ozaf017-B36] Mariño-Ramírez L, Kann MG, Shoemaker BA & Landsman D (2005). Histone structure and nucleosome stability. Expert Rev Proteomics 2(5), 719–729. 10.1586/14789450.2.5.719 16209651 PMC1831843

[ozaf017-B37] Masiello I & Biggiogera M (2017). Ultrastructural localization of 5-methylcytosine on DNA and RNA. Cell Mol Life Sci 74(16), 3057–3064. 10.1007/s00018-017-2521-1 28391361 PMC11107537

[ozaf017-B38] Moore LD, Le T & Fan G (2013). DNA methylation and its basic function. Neuropsychopharmacology 38(1), 23–38. 10.1038/npp.2012.112 22781841 PMC3521964

[ozaf017-B39] Morris KV & Mattick JS (2014). The rise of regulatory RNA. Nat Rev Genet 15(6), 423–437. 10.1038/nrg3722 24776770 PMC4314111

[ozaf017-B40] Musselman CA, Lalonde M-E, Côté J & Kutateladze TG (2012). Perceiving the epigenetic landscape through histone readers. Nat Struct Mol Biol 19(12), 1218–1227. 10.1038/nsmb.2436 23211769 PMC3645987

[ozaf017-B41] Penczek PA (2010). Resolution measures in molecular electron microscopy. Methods Enzymol 482, 73–100. 10.1016/S0076-6879(10)82003-8 20888958 PMC3165049

[ozaf017-B42] Pichon X, Lagha M, Mueller F & Bertrand E (2018). A growing toolbox to image gene expression in single cells: Sensitive approaches for demanding challenges. Mol Cell 71(3), 468–480. 10.1016/j.molcel.2018.07.022 30075145

[ozaf017-B43] Pogribny IP & Beland FA (2009). DNA hypomethylation in the origin and pathogenesis of human diseases. Cell Mol Life Sci 66(14), 2249–2261. 10.1007/s00018-009-0015-5 19326048 PMC11115809

[ozaf017-B44] Prakash K, Fournier D, Redl S, Best G, Borsos M, Tiwari VK, Tachibana-Konwalski K, Ketting RF, Parekh SH, Cremer C & Birk UJ (2015). Superresolution imaging reveals structurally distinct periodic patterns of chromatin along pachytene chromosomes. Proc Natl Acad Sci U S A 112(47), 14635–14640. 10.1073/pnas.1516928112 26561583 PMC4664314

[ozaf017-B45] Sarmento MJ, Oneto M, Pelicci S, Pesce L, Scipioni L, Faretta M, Furia L, Dellino GI, Pelicci PG, Bianchini P, Diaspro A & Lanzanò L (2018). Exploiting the tunability of stimulated emission depletion microscopy for super-resolution imaging of nuclear structures. Nat Commun 9(1), 3415. 10.1038/s41467-018-05963-2 30143630 PMC6109149

[ozaf017-B46] Schermelleh L, Ferrand A, Huser T, Eggeling C, Sauer M, Biehlmaier O & Drummen GPC (2019). Super-resolution microscopy demystified. Nat Cell Biol 21(1), 72–84. 10.1038/s41556-018-0251-8 30602772

[ozaf017-B47] Schmidt R, Weihs T, Wurm CA, Jansen I, Rehman J, Sahl SJ & Hell SW (2021). MINFLUX nanometer-scale 3D imaging and microsecond-range tracking on a common fluorescence microscope. Nat Commun 12(1), 1478. 10.1038/s41467-021-21652-z 33674570 PMC7935904

[ozaf017-B48] Shakoori AR (2017). Fluorescence in situ hybridization (FISH) and its applications. Chromosome Structure and Aberrations 1, 343–367. 10.1007/978-81-322-3673-3_16

[ozaf017-B49] Shatskikh AS, Kotov AA, Adashev VE, Bazylev SS & Olenina LV (2020). Functional significance of satellite DNAs: Insights from Drosophila. Front Cell Dev Biol 8, 312. 10.3389/fcell.2020.00312 32432114 PMC7214746

[ozaf017-B50] Shrestha D, Jenei A, Nagy P, Vereb G & Szöllősi J (2015). Understanding FRET as a research tool for cellular studies. Int J Mol Sci 16(4), 6718. 10.3390/ijms16046718 25815593 PMC4424985

[ozaf017-B51] Sinefeld D, Xia F, Wang M, Wang T, Wu C, Yang X, Paudel HP, Ouzounov DG, Bifano TG & Xu C (2022). Three-photon adaptive optics for mouse brain imaging. Front Neurosci 16, 880859. 10.3389/fnins.2022.880859 35692424 PMC9185169

[ozaf017-B52] Stepanov AI, Besedovskaia ZV, Moshareva MA, Lukyanov KA & Putlyaeva LV (2022). Studying chromatin epigenetics with fluorescence microscopy. Int J Mol Sci 23(16), 8988. 10.3390/ijms23168988 36012253 PMC9409072

[ozaf017-B53] Tekle YI, Wang F, Heidari A & Stewart AJ (2020). Differential gene expression analysis and cytological evidence reveal a sexual stage of an amoeba with multiparental cellular and nuclear fusion. PLoS One 15(11), e0235725. 10.1371/journal.pone.0235725 33147262 PMC7641356

[ozaf017-B54] Tekle YI, Wood FC, Katz LA, Cerón-Romero MA & Gorfu LA (2017). Amoebozoans are secretly but ancestrally sexual: Evidence for sex genes and potential novel crossover pathways in diverse groups of amoebae. Genome Biol Evol, 9(2):375–387. 10.1093/gbe/evx002 28087686 PMC5381635

[ozaf017-B55] Thorn K (2016). A quick guide to light microscopy in cell biology. Mol Biol Cell 27(2), 219–222. 10.1091/mbc.E15-02-0088 26768859 PMC4713126

[ozaf017-B56] Wang Y, Eddison M, Fleishman G, Weigert M, Xu S, Wang T, Rokicki K, Goina C, Henry FE, Lemire AL, Schmidt U, Yang H, Svoboda K, Myers EW, Saalfeld S, Korff W, Sternson SM & Tillberg PW (2021). EASI-FISH for thick tissue defines lateral hypothalamus spatio-molecular organization. Cell 184(26), 6361–6377.e24. 10.1016/j.cell.2021.11.024 34875226

[ozaf017-B57] Wang X & Lai Y (2021). Three basic types of fluorescence microscopy and recent improvement. E3S Web Conf 290, 01031. 10.1051/e3sconf/202129001031

[ozaf017-B58] Weinhold B (2006). Epigenetics: The science of change. Environ Health Perspect 114(3), A160–A167. 10.1289/ehp.114-a160 16507447 PMC1392256

[ozaf017-B59] Wills M. (2018). The Evolution of the Microscope. JSTOR Daily. https://daily.jstor.org/the-evolution-of-the-microscope/ (retrieved March 16, 2025).

[ozaf017-B60] Xie Y, Habibalahi A, Anwer AG, Wahi K, Gatt C, Johansson EMV, Holst J, Goldys E, & Zanini F. (2024). Integration of hyperspectral imaging and transcriptomics from individual cells with HyperSeq. bioRxiv 577536. 10.1101/2024.01.27.577536 PMC12315715 40628527

[ozaf017-B61] Yang KD, Belyaeva A, Venkatachalapathy S, Damodaran K, Katcoff A, Radhakrishnan A, Shivashankar GV & Uhler C (2021). Multi-domain translation between single-cell imaging and sequencing data using autoencoders. Nat Commun 12(1), 31. 10.1038/s41467-020-20249-2 33397893 PMC7782789

[ozaf017-B62] Young AP, Jackson DJ & Wyeth RC (2020). A technical review and guide to RNA fluorescence in situ hybridization. PeerJ 8, e8806. 10.7717/peerj.8806 32219032 PMC7085896

[ozaf017-B63] Yue M, Richard JLC, Yamada N, Ogawa A & Ogawa Y (2014). Quick fluorescent in situ hybridization protocol for Xist RNA combined with immunofluorescence of histone modification in X-chromosome inactivation. J Vis Exp 93, e52053. 10.3791/52053 PMC4354415 25489864

[ozaf017-B64] Zhang X, Wang L, Li N & Xiao Y (2021). Assessing chromatin condensation for epigenetics with a DNA-targeting sensor by FRET and FLIM techniques. Chin Chem Lett 32(8), 2395–2399. 10.1016/j.cclet.2021.02.031

